# P11. Developing an immunotherapy strategy for the effective treatment of patients with Non Small Cell Lung Cancer (NSCLC): strategies to evaluate immunity in patients on clinical trials

**DOI:** 10.1186/2051-1426-2-S2-P2

**Published:** 2014-03-12

**Authors:** M Neuberger, C Paustian, T Hilton, N Engel, S Reu, A Linder, R Huber, R Hatz, H Winter

**Affiliations:** 1 Klinikum der Universität München, Klinik für Chirurgie, Munich, Germany; 2 Robert W. Franz Cancer Center, Earle A. Chiles Research Institute, Providence Portland Medical Center, Laboratory of Molecular and Tumor Immunology, Portland, OR, USA; 3 UbiVac, Portland, OR, USA; 4 Ludwig-Maximilians-University of Munich, Department of General and Thoracic Surgery, Grosshadern, Germany; 5 Ludwig-Maximilians-University of Munich, Institute of Pathology, Munich, Germany; 6 Asklepios Fachkliniken-München-Gauting, Department of Thoracic Surgery, Gauting, Germany; 7 University of Munich, Division of Pneumology, Department of Medicine Klinikum Innenstadt, Munich, Germany; 8 Robert W. Franz Cancer Center, Earle A. Chiles Research Institute, Providence Portland Medical Center, Portland, OR, USA

## Background

Our centers have formed an international collaborative group composed of surgeons, pathologists, radiation oncologists, medical oncologists and immunologists working together with biotech and pharma sectors to evaluate biomarkers of immune status and develop effective combination immunotherapy for patients with NSCLC.

## Material and methods

Two phase I immunotherapies for NSCLC following surgical resection were begun at our institutions. One involved stage I-IIIA patients given an irradiated, autologous whole-tumour cell vaccine following induction of lymphopenia by chemotherapy and reinfusion of autologous peripheral blood mononuclear cells (PBMC). The other recruited stage IV patients given an autophagosome-enriched vaccine generated from irradiated autologous tumour cells. Histologic sections, enzymatically-digested tumour, pleural effusions and apheresis are available from a number of these patients. Current efforts are evaluating immunoscore and immune profiling by IHC and comparing results with flow cytometric analyses of the tumour.

## Results

Preliminary studies have evaluated CD3+ CD8+ CD45RO+ cytotoxic memory T cells associated with tumour. We are continuing to examine myeloid to lymphoid infiltrate ratios. While preliminary, and with only 4 patients evaluated, preexisting autologous tumour-reactive T cells (IFN-γ) were only detected when T cells predominated in the tumour preparation analysed. Studies comparing this with IHC staining and analysis using Definiens software are underway.

## Conclusions

A consortium of institutions has come together to improve the outcome of patients with NSCLC. The progress made to date will be used to evaluate immune responses to next generation immunotherapy. Evaluation of gene expression profiling of NSCLC has identified common overexpressed antigens and an off the shelf vaccine of autophagosomes containing at least 9 NCI prioritised cancer antigens, 5 TLR agonists and a DC targeting molecule has been developed. A multi-center phase II trial of combination immunotherapy for NSCLC employing this vaccine is open in the USA and efforts are underway to open this trial in Munich with coordinated efforts to evaluate anti-cancer immunity in patients on this and other trials.

